# Activated Charge-Reversal Polymeric Nano-System: The Promising Strategy in Drug Delivery for Cancer Therapy

**DOI:** 10.3390/polym8040099

**Published:** 2016-04-05

**Authors:** Yichen Hu, Xiao Gong, Jinming Zhang, Fengqian Chen, Chaomei Fu, Peng Li, Liang Zou, Gang Zhao

**Affiliations:** 1 School of Pharmacy and Bioengineering, Chengdu University, Chengdu 610106, China; huyichen0323@126.com (Y.H.); zouliang@cdu.edu.cn (L.Z.); 2 State Key Laboratory of Silicate Materials for Architectures, Wuhan University of Technology, Wuhan 430070, China; 3 School of Pharmacy, Chengdu University of Traditional Chinese Medicine, Chengdu 611137, China; zhangjinming1987@126.com (J.Z.); chaomeifu@126.com (C.F.); 4 State Key Laboratory of Quality Research in Chinese Medicine, Institute of Chinese Medical Sciences, University of Macau, Macao 999078, China; PengLi@umac.mo; 5 Department of Microbiology & Immunology, MCV Campus School of Medicine, Virginia Commonwealth University, Richmond, VA 23284, USA; chenf3@vcu.edu

**Keywords:** polymeric nano-carriers, polyelectrolytes, drug delivery, surface charge, cellular uptake

## Abstract

Various polymeric nanoparticles (NPs) with optimal size, tumor-targeting functionalization, or microenvironment sensitive characteristics have been designed to solve several limitations of conventional chemotherapy. Nano-sized polymeric drug carrier systems have remarkably great advantages in drug delivery and cancer therapy, which are still plagued with severe deficiencies, especially insufficient cellular uptake. Recently, surface charge of medical NPs has been demonstrated to play an important role in cellular uptake. NPs with positive charge show higher affinity to anionic cell membranes such that with more efficient cellular internalization, but otherwise cause severe aggregation and fast clearance in circulation. Thus, surface charge-reversal NPs, specifically activated at the tumor site, have shown to elegantly resolve the enhanced cellular uptake in cancer cells *vs.* non-specific protein adsorption dilemma. Herein, this review mainly focuses on the effect of tumor-site activated surface charge reversal NPs on tumor treatment, including the activated mechanisms and various applications in suppressing cancer cells, killing cancer stem cell and overcoming multidrug resistance, with the emphasis on recent research in these fields. With the comprehensive and in-depth understanding of the activated surface charge reversal NPs, this approach might arouse great interest of scientific research on enhanced efficient polymeric nano-carriers in cancer therapy.

## 1. Introduction

Cancer is one of the world’s most fatal diseases with more than 10 million new cases and eight million related deaths every year [1,2]. Chemotherapy is one of the major treatment approaches to offset the inapplicability of surgery [3,4]. However, for more than two decades, advances in understanding cancer biology still have not translated into anticancer success. Major anticancer drugs are confronted with short circulation period and insufficient localization to tumor sites [5,6]. Even more troubling issues than this problem are drug toxicity and tumor resistance. These anticancer drugs often also injure healthy cells and cause toxicity to patients, which causes major complications, such as neutropenia syndrome or serious heart failure [7,8]. It would necessitate cessation of administration. Furthermore, drug resistance continues to be a major obstacle [9,10]. Given these issues associated with cancer treatment safety and efficacy, increasingly researchers are paying great attention to utilizing nanomedicines in the fight against cancer.

Over the past decades, nanotechnology-based therapeutics have exhibited clear benefits in cancer diagnostics, prevention, and treatment. Chemotherapy based on nanomedicine, an offshoot of nanotechnology, has been demonstrated to possess improved half-life, retention period, tumor targeting efficiency, and fewer patient side effects, compared with that of unmodified drugs [11]. A number of nanomedicines including liposomes, polymeric carriers, dendrimers, and inorganics as well as others have been employed for the development of new cancer therapeutics [12,13]. These nanocarriers are used to improve bioavailability and/or selectivity of anticancer drugs via prolonging circulation and EPR effect [14]. Up to today, about 250 nano-scaled drug delivery products have been developed in various stages of preclinical and clinical process [15,16]. Doxil [17], Abraxane [18] and Genexol-PM [19] are successful examples of NPs approved for clinical applications. On the preclinical front, several nanomaterial formulations using silica, polymer, metal, and carbon-based materials, with both active and passive targeting properties, have shown promise [20].

Thus far, however, the clinical outcome of these nano-scaled drug formulations is relatively disappointing, failing to improve response rates and survival times [21]. One major limitation is their insufficient cellular uptake owing to a stealth water-soluble surface like poly(ethylene glycol) (PEG) or poly(ethylene oxide) (PEO) [22]. Although the hydrophilic layer could prolong circulation time and enhance tumor accumulations by improved permeability and retention (EPR) effect, it fails to create optimal cellular uptake of NPs in tumor sites [23]. Recently, this problem, referred to as the “PEG dilemma”, has been widely improved by the addition of targeting ligands on the outer shell of NPs to specifically recognize the over-expressed receptors in tumor cells [24]. Various ligands such as antibodies, aptamers, proteins, peptides, folate, carbohydrate and other emerging targeting molecules modified on NPs have been developed [25,26,27,28] to facilitate NP–cell binding, entering cells via the receptor-mediated endocytic route and the release of drugs effectively.

However, the arrival of these ligand modified NPs directly to tumor cells still might be hindered by the uncontrollable and volatile receptor levels owing to tumor heterogeneity [29]. This shortage is further frustrated by the elevated interstitial pressure of tumors owing to the leaky nature of their blood vessels and their dysfunctional lymphatic vessels [30], which can impede NPs tumor penetration and retention [21]. To date, surface charge has been generally demonstrated to affect the efficiency of cellular uptake for miscellaneous NPs by adjusting the adhesion of NPs and their interactions with cells [31,32]. This review will focus on the recent progress of these activated charge-reversal NPs for cancer therapy.

## 2. Role of Surface Charge on Cellular Uptake

Initially, before discussing the application of activated charge-reversal NPs, we would like to briefly introduce the role of surface charge of NPs on cellular uptake, which could provide thorough evidence for the reason to develop these NPs with charge-conversion. Cellular uptake is the critical factor for intracellular drug delivery, among which surface charge of NPs plays a key role [33]. As mentioned above, these neutral or negatively charged NPs with PEG layer encounter poor cellular uptake. In contrast to NPs with neutral or negative charge, NPs with positive charge show significantly higher affinity with negatively charged phospholipid head groups, glycans as well as proteins on cell membranes, and have been more efficient in penetration of cell-membrane and cellular internalization in many previous investigations [34,35,36]. Chen *et al.* [37] showed that more positively charged hydroxyapatite (HAP) NPs could penetrate into osteoblast cells compared with those negatively charged NPs with similar size and shape, which is attributed to the attractive or repulsive interaction between cell membranes and different charged NPs. Additionally, compared to receptor-mediated uptake, the charged surface exhibits another advantage that positively charged NPs would expand the particle size limitation, due to the limited radius of cell membrane, for appropriate uptake of NPs. Gao’s study [38] showed that negatively charged NPs have smaller optimal wrapping size (˂200 nm) than positively charged NPs with higher size of 320 nm.

Moreover, the exposed charge also significantly affects the cellular uptake mechanism, including clathrin- and caveolae-mediated or independent endocytosis, and marcopinocytosis [39,40,41]. Most negatively charged NPs illustrated an inferior rate of endocytosis, while NPs with positive charge can be internalized by clathrin-mediated endocytosis [42]. Positively charged NPs composed of polycationic polymers like poly(ethyleneimine) can cross the cell membrane directly [43]. Nevertheless, the use of positively charged NPs is unfortunately limited by their side-effects, *i.e.*, the strong interactions with serum components in blood and blind cytotoxicity against normal cells [44,45]. These superfluous functions would lead to rapid clearance, hemolytic side effect and toxicity to normal tissue [46]; hence, these cationic NPs, despite higher cellular uptake, are not favorable as drug carriers for *in vivo* applications. Thus, the fabrication of “charge-reversal” NPs with negative charge in blood circulation, but altering to positive charge in tumor site for selectively improving the internalization by tumor cells is highly anticipated [47].

## 3. Activated Approaches of Surface Charge Reversal

**Figure 1:**
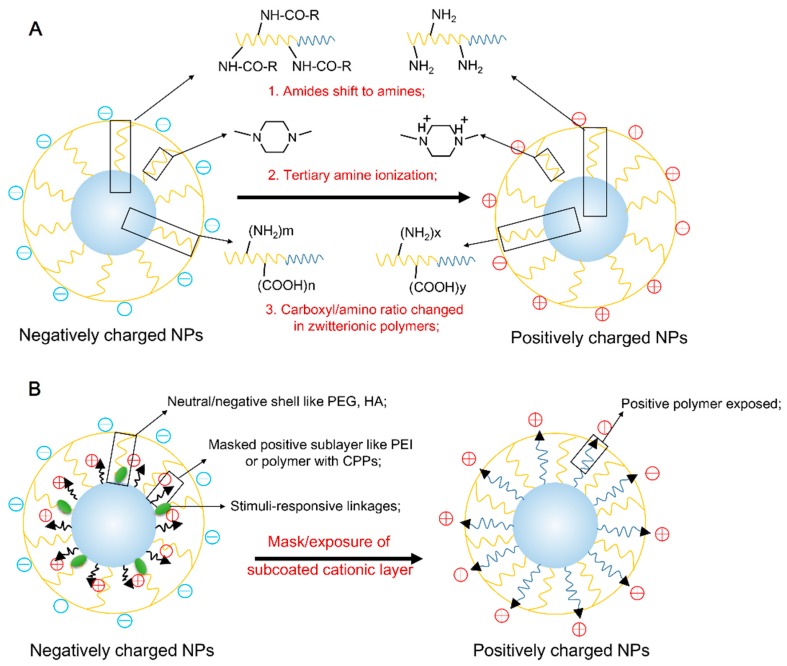
Mechanisms of activated charge reversal by protonation/deprotonation of polymer (**A**) and mask/exposure of subcoated cationic nano-layer (**B**).

Recently, the “negative-to-positive charge reversal” approach is achieved by the activation of representative typical stimuli include pH-shift, redox reaction, enzyme, ultraviolet and temperature (Table 1). Of these stimuli, pH-responsive charge reversal is most frequently employed because of obvious pH distinction between that in physiological environment and tumor site. For example, the extracellular environment of tumor is more acidic (pHe 6.5~7.2) than normal tissue and blood (pH ≈ 7.4), with the pH value further decreasing to lower than 6.0 in endosome and lysosome. These ideal drug delivery systems specifically reach the desired target site and bypass the normal tissues by the “Trojan horse” behavior. On this occasion, the cationic NPs are first masked at the physiological pH to avoid premature clearance in intravenous *(i.v.)* administration, but once they are exposed to these intrinsic/extrinsic stimuli, the NPs’ positive charge is recovered. The activated charge-reversal NPs have the ability to combine the advantage of positive and negative charge, improving the cellular uptake efficiency. Generally, these activated charge-reverse NPs are achieved by two approaches: (i) protonation/deprotonation of polymer; and (ii) mask/exposure of subcoated cationic nano-layer (Figure 1).

### 3.1. The Stimuli-Responsive Protonation/Deprotonation of Polymer

Particularly, tumor pHe is the important trigger for the protonation/deprotonation conversion of polymers. Many previous reports have given strong evidence of the feasibility of tumoral pHe activated charge-reversal strategy. Commonly, the charge conversion in the majority of this kind of pH-responsive polymers results from the transformation of prototypical amine into amide. Xu *et al.* [48] demonstrated the charge-reversal concept that amide groups exhibit pH-dependent hydrolysis. Polycaprolactone (PCL)-*block*-PEI was synthesized, in which 20% of the primary and secondary amines of PEI was preserved by amides with 1,2-cyclohexanedicarboxylic anhydride. The amide derived from both the secondary and primary amine will be readily hydrolyzed at pH 5 and pH 6, but only 50% even after 60 h at pH 7.4. Therefore, the negatively charged PCL-PEI/amide micelles exhibited a zeta potential of −20 mV at pH 7.4 after over 60 h, while they gradually became positively charged at pH 6 and even reached +50 mV at pH 5. Similar to the activation mechanism adopted by this way of the functionalized terminal amino of poly(2-aminoethyl methacrylate hydrochloride) (PAMA) masked with 2,3-dimethylmaleic anhydride (DMMA), the pHe-triggered charge-reversal nanogel [49] was fabricated, in which the resultant amide bond was more stable at neutral and alkali pH values, but cracked promptly in response to tumor pHe and exposed positive-charge amino groups again. Interestingly, the PAMA-DMMA nanogel with charge conversion activity could alleviate protein absorption at pH 7.4 while interacting strongly with tumor cells under slightly acidic conditions. In view of the advantage of amide groups, several tumoral pHe activated charge-reversal nano-carriers have been developed recently, as shown in Table 1.

Besides the amide shift approach mentioned, the protonation/deprotonation conversion of polymers was also achieved in some other ways. Poly(β-amino ester) (PBAE) [50], a cationic polymers containing tertiary amines, has been utilized as pH-responsive drug carriers. Currently, micelles formed by amphiphilic polymers with PBAE grafting hydrophilic block like PEG could possess acidic-triggered drug release, by the protonation activity of tertiary amine in PBAE blocks [51]. Interestingly, the charge reversion of PBAE polymer from neutral/anionic to cationic was observed [52]. Chen *et al.* developed a new d-α-tocopheryl polyethylene glycol 1000-poly(β-amino ester) block copolymer for docetaxel loading, which formed stable micelles with negative charge in physiological pH. Decrease in pH could cause the increase of hydrophilicity of these polymers as well as the charge reverse. When pH decreased from 7.4 to 6.5 in order to mimic the pHe variation, its pH-dependent surface charge reversal of zeta potential from −47.6 ± 2.5 mV to +22.5 ± 3.2 mV were pleasantly found. 

In addition, the pHe-sensitive charge-reversal has also been achieved in zwitterionic NPs by regulating the ratio of amino and carboxyl group in polymers instead of modifying or masking groups. Poly(l-glutamic acid) and poly(l-lysine), as the typical acidic and alkali polypeptides, respectively, have many side carboxyl and amino groups, so that different ratio in their complex could result in different charge. In a previous study, the pH-responsive behavior of poly (*N*-isopropylacrylamide)-*block*-poly (l-glutamic acid-*co*-l-lysine) PNiPAM (PLG-*co*-PLLys) copolymer has been demonstrated by controlling their ratio and the following protonation and deprotonation competition [53]. Thus, Huang *et al.* [54] reported a charge-reversal poly(l-glutamic acid-*co*-l-lysine) nano-aggregates to regulate the ratio of pH-sensitive side groups of amino and carboxyl group in polymers. A series of poly(l-glutamic acid-*co*-l-lysine) copolymers with different feed ratio of l-glutamic acid and l-lysine ratio (4.1/1, 1.5/1, 0.97/1, and 1/1.5) were synthesized to regulate the protonation or deprotonation degree of NPs in acidic environment (6.5–7.2). The result evidenced that by manipulating cisplatin loading rate, surface charge of zwitterionic NPs could be reversed from negative to positive at tumor pHe.

### 3.2. The Stimuli-Responsive Mask/Exposure of Subcoated Cationic Nano-Layer

Apart from the ionization of groups in polymers, the stimuli-responsive steric hindrance is another common way to mask the cationic profile of NPs as the “*Trojan horse*” delivery approach. The system with steric hindrance exhibits a “shell-core” structure, consisting of: (a) the neutral/negative polymeric shell to create steric coating over the subcoat; and (b) the cationic polymeric core with cargo loading. On the one hand, PEG shielding is the most effective approach to create steric hindrance and improve long-circulation effect. Various PEGylation NPs can prolong the circulation time, improving their preferential accumulation in tumor site via EPR effect. However, PEG shell can severely impede tumor uptake. On the other hand, in many previous studies the positive-charge polymeric core is generally resulted from the anchored cationic cell penetrating peptides (CPP) like HIV-transactivator (TAT) peptide or PEI polymer. TAT peptide which is derived from human immunodeficiency viruses types 1 and 2 (HIV-1 and HIV-2) is one of non-specific CPP, and serves to quickly enter into cells both *in vitro* and *in vivo* [55,56]. Although the precise entry pathways are still controversial, its greatly cationic activity is likely to be the reliable mechanism to internalize into cells by passive electrostatic interactions. Nevertheless, the most important stratagem in this system to achieve the transition from negative to positive charge is the stimuli-triggered re-emergence of cationic core by shielding/deshielding transitions.

A typical design is on the basis of the “long and short chains” model. According to the model, a long-chain PEG with or without targeting ligands is employed to cover the short-chain core with CPP. PEG shell would be divorced with the bond cleavage in response to the specific stimuli including tumor pHe, matrix metallo proteinases (MMP), and high concentration of glutathione. The function of CPPs will be reinstalled when they are fully exposed on the carrier surface. A proof-of-concept study was reported in a study about pHe-triggered TAT exposure micelle system. Bae’s group [57] fabricated a mixed polymeric micelle composing by two components, TAT peptide conjugated PEG-*b*-PLLA and PEG-*b*-poly(methacryloyl sulfadimethoxine) (PSD-*b*-PEG). At pH 7.4, PSD is negatively charged and mask the cationic TAT on micelle surfaces via electrostatic interaction. The addition of outer PEG layer also can shield TAT peptide. However, when pH decreased in tumor acidic region (about 6.6), the PSD part turned to the unionized form and detached itself from the cationic TAT, which led to reactivate TAT-mediated cell penetration. Additionally, Bae *et al.* [58] also developed a different strategy involves a “shielding and exposure” process based on the “pop-up” mechanism. This kind of system was involved in two block copolymers: PLA_3k_-*b*-PEG_2k_-*b*-polyHis_2k_-TAT and polyHis_5k_-*b*-PEG_3.4k_. Initially, due to the non-ionization of polyHis_2k_ at neutral pH, poly His_2k_-TAT was designed to hide in the core of mixed micelles formed by hydrophobic parts (PLA_3k_ and polyHis_5k_ blocks). Being shielded by a hydrophilic corona of PEG_2k_ and PEG_3.4k_ blocks, the cationic TAT was therefore masked by the long-chain PEG block. Due to the pH-responsive ionization process, the hydrophobic polyHis_2k_ transformed into hydrophilic block, and caused the TAT peptide to pop out of the surface, for the reason of its longer hydrophilic polymeric block than PEG_3.4k_. Thereby, the shell of micelles became positively charged at acidic pHe, facilitating cellular internalization and endosome escape. By exposure of TAT on the surface of these micelles at acid value, its uptake was increased by 30-fold and even up to 70-fold at pH 7.0 and 6.8, respectively, compared to that at pH 7.4.

Furthermore, the expression of some enzymes in tumor extracellular matrix is up-regulated in a range of tumors. Therefore, enzymatic deshielding based on protease-sensitive linker shows an ideal approach to specifically expose the cationic layer in tumor. Hyaluronidase (HAase) [59,60], the major enzyme rich in the tumor microenvironment and the tumoral cellular endosomes and lysosomes, could efficiently degrade hyaluronic acid (HA) [61,62], a natural anionic mucopolysaccharide. Tian *et al.* [63] fabricated HA coated polyethylenimine-poly(γ-benzyl l-glutamate)/DNA (PEI-PBLG/DNA) complexes with a multiple-layer structure to attain the goal of tumoral enzyme-triggered charge-reversal. HA can be flexibly coated on the cationic surface of polymer gene carriers to counteract its positive charge by electrostatic interaction. HA coating can reduce the electrostatic binding affinity of PEI-PBLG/DNA to cells, while enhances the transfection efficiency of PEI-PBLG along with the degradation of HA coating in cells. Moreover, the overexpressed extracellular matrix metalloprotease2 (MMP2) [64] in tumor tissue is also the effective trigger for shield/deshield transition. The MMP2 substrates including cleavable peptides have been shown as MMP responsive degradable in previous studies [65,66]. Torchilin’s group [67] employed an MMP2-cleavable octapeptide (Gly-Pro-Leu-Gly-Ile-Ala-Gly-Gln) as the cleavable linker in a “long and short chain” liposome system composing by mAb 2C5-PEG_3.4k_-MMP2 peptide-DOPE and TAT-PEG_2k_-DSPE. The long-chain PEG_3.4k_ block worked as a steric shield for NPs and surface-attached TAT in the blood. Nevertheless, the cleavage of the peptide by the highly expressed extracellular MMP2 in the tumor microenvironment resulted in the exposure of cationic NPs with TAT functionalized and the enhanced intracellular penetration.

Similarly, He’s group [68] utilized the steric hindrance of thiolytic-cleavable PEG for the mask of TAT-conjugated liposomal core, in response to the high reductive tumor microenvironment. They also chased this aim with the aid of “long and short chain” strategy. TAT conjugated on a short-chain PEG-lipid (DOPE-PEG_1.6k_-TAT) was also shielded by the long-chain PEG_5k_-lipid with redox-responsive disulfide linker (DOPE-S-S-mPEG_5k_). By this mean, cleavage of the disulfide bond in tumoral redox-environment would result in cleavage of the long-chain PEG and detachment, to expose TAT-linked liposome. Results showed that cellular uptake of liposome with addition of exogenous l-Cys would dramatically increase by three fold in comparison of that in physiological environment by the PEG steric effect.

Other than application of the intrinsic stimuli, the method of utilizing extrinsic stimuli like thermo- and UV-sensitive charge-reversion was also investigated. A mild thermal stimulus used to control “shielding/deshielding” of CPP-modified liposomes to facilitate drug delivery was reported [69]. The thermosensitive poly (*N*-iso-propylacrylamide) (PNIPAAm), with a lower critical solution temperature (LCST) behavior around 32 °C, was linked on the liposomes to mask the immobilized CPPs on the surface of carriers. PNIPAAm chains were hydrated and elongated to shield CPPs below the LCST, while dehydrated and aggregated with higher temperature above it. It could stretch or shrink with the temperature shift to activate CPPs and achieve the surface charge reversal. A comparable study with heat-activable cationic liposomes with CPPs exposure was given by Mei’s group [70]. Thermosensitive liposomes (TSL), in which lipid materials will have a phase transition upon heating in a mild-hyperthermia range, were fabricated to cover CPP-DOX conjugate. As a result, the zeta potential of CPP-DOX/TSL pre-heated and that after heating at 42 °C for 30 min was −21.8 mV and +4.12 mV, resulting in enhancing intracellular drug delivery of the liposomes to HT-1080 cells. Otherwise, Hansen *et al.* [71] developed an UV-activatable charge-reversal liposomal system with TAT anchored on liposomal surface with a constrained and deactivated form as the loop structure (“n” shape). An UV-cleavable alkyl anchor was designed to conjugate TAT and a PEG-phospholipid, appearing TAT overclouded by PEG chain. In this way, the TAT modified liposomes with a negative zeta-potential could transformed into positive-charged with revived cell penetration ability under UV irradiation.

## 4. Activated Surface Charge Reversal NPs for Cancer Treatment

With the increasing attention on pros and cons of positive and negative charge, the development of diversified NPs with a charge-reversal concept has become an important issue for improved anticancer efficiency, to latently deactivate surface positive charge in blood circulation, but reactivate once inside the targeted tissues or cells. Abundant studies have demonstrated the success of this concept with better therapeutic outcome.

### 4.1. Enhanced Cellular Uptake and Intracellular Delivery

As is well known, neutral polymers such as PEG, poly(ethylene oxide) and poly(acrylic acid) could protect anticancer agents from being cleared *in vivo* with a long circulation time and high drug accumulation in tumor sits via EPR effect [94]. But to be honest, only sufficient concentration of drugs entering in target tumor cells bring the efficient anticancer activity. Unfortunately, the dense polymer layer meanwhile prohibits the tumoral cellular internalization of anticancer drugs, resulting in insufficient anticancer outcome [24]. The process of cellular uptake of NPs is usually viewed as two steps: firstly, the binding step on cell membranes and secondly, the internalization step [95]. Based on the negatively charged sulfated proteoglycans in cellular surface, NPs with positive charge could bind strongly to cell membrane by electrostatic interactions, following the several possible internalization mechanisms [41,96,97]. Thus, the activated charge-reversal strategy provides a promising way for cancer therapy to accomplish rapid cellular uptake with carried cargoes at the pathological site, maximizing the anticancer efficacy.

Some investigations have shown the role of surface charge-reversal NPs on cellular uptake in this regard. For example, based on the zeta potential transition from pH 7.4 to pH 6.8 of CDDP/P(Glu-*co*-Lys) NPs, the remarkable pH-relevant uptake and cytotoxicity behavior was observed in Hela cells [54]. Wang and his group [49] designed an acidic activated charge-reversal PAMA nanogel with amino groups deactivated by DMMA, and compared its internalization behavior at pH 7.4 and pH 6.8 by confocal laser scanning microscopy (CLSM) and fluorescence-activated cell sorting (FACS). Being labeled with FITC, PAMA-DMMA nanogel could be clearly observed to internalize into cells to a significant extent and distribute extensively in the cytoplasm at pH 6.8, whereas it seemed to be mainly attached to the MDA-MB-435s cell membrane at pH 7.4. This observation result was further confirmed by FACS data, in which remarkably enhanced intracellular fluorescence was detected at pH 6.8 in comparison to that at pH 7.4. Importantly, there was no difference on cellular uptake of the non-charge-conversional PAMA-SA nanogel at between pH 7.4 and 6.8 when it was incubated with cells in like manner. Accordingly, with DOX loading, PAMA–DMMA nanogel at pH 6.8 showed significantly enhanced *in vitro* cytotoxicity relative to at pH 7.4 (*p* < 0.005), with 57% and 30% cell viability at pH 7.4 and 6.8, respectively, at the same loaded DOX concentration of 16 μg/mL^−1^, respectively. The charge-conversional strategy for cancer therapy further exhibited the advantages in tumoral distribution of tumor-bearing Balb/c-nu mice. With the enhanced cellular uptake activity, PAMA-DMMA nanogel seemed to possess higher cellular concentration in cytoplasm, whereas PAMA-SA nanogel mainly remained in the extracellular space and/or adhered to cell membranes.

Otherwise, Wang *et al.* [75] also fabricated surface charge switchable NPs based on zwitterionic polymers, *i.e.*, PCL-*b*-P(AEP-*g*-TMA/DMA), containing tertiary amine and carboxy group in pH-sensitive amides. Based on about −3 mV and +20 mV of sensitive charge-reversal NPs at pH 7.4 and pH 6.8 at 2 h, respectively, the *in vitro* cellular uptake of DOX-loaded NPs at pH 6.8 was significantly higher in MDA-MB-231 cells after 2 h incubation than that at pH 7.4. Furthermore, compared to either free DOX or insensitive NPs, the higher tumoral cellular uptake of activatable NP was also observed in a breast cancer bearing nude mouse model. It supported the proposal design that charge switchable NPs could respond to the acidic tumor environment, switching to positive charge, and facilitating the tumor cellular uptake as well as enhanced accumulation in tumor tissues.

**Figure 2:**
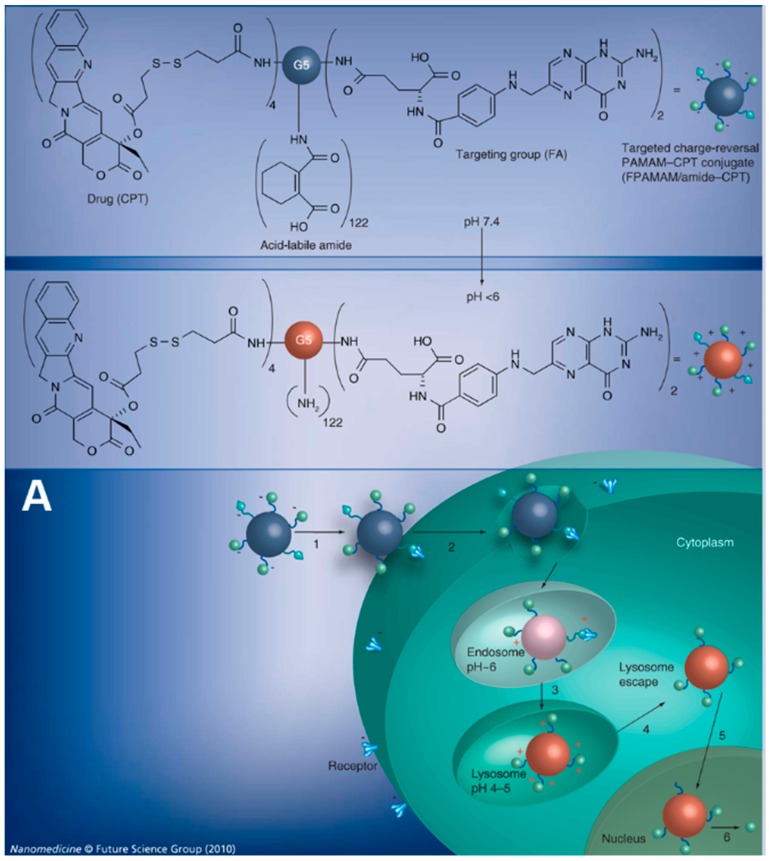

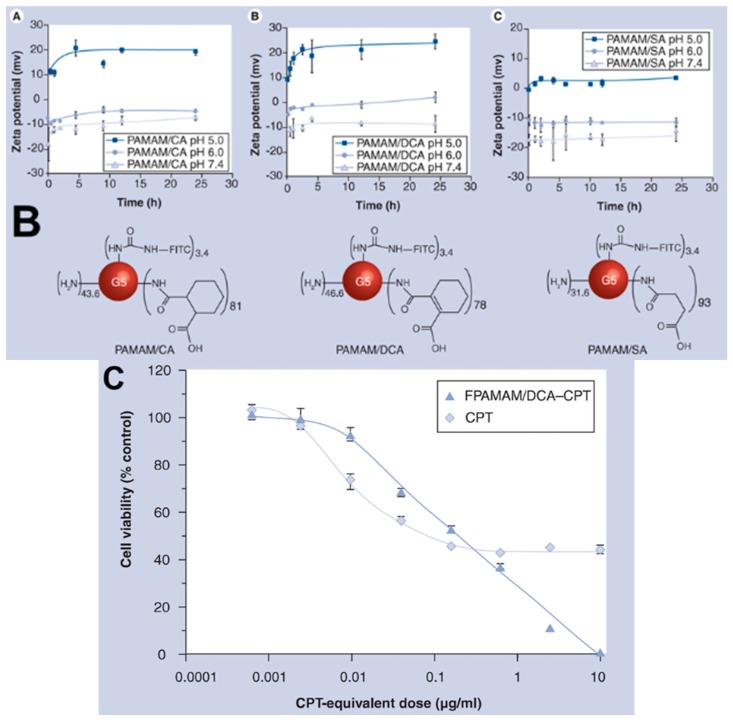
Charge-reversal polyamidoamine NPs for cascade nuclear delivery: (**A**) scheme of charge-reversal mediated intracellular drug delivery; (**B**) the acid-trigged charge reversal from negative to positive; and (**C**) the cytotoxicity against SKOV-3 ovarian cancer cells for 24 h treatment. Note: Folic receptor-targeting charge-reversal PAMAM–CPT conjugate (FPAMAM/amide–CPT); Trans-1,2-cyclohexanedicarboxylic anhydride(CA); 1,2-dicarboxylic-cyclohexene anhydride(DCA); Succinic anhydride(SA). Reproduced with permission from Shen *et al.* (2010). Copyright 2010 Future Medicine.

Furthermore, apart from the improved internalization process, charge-reversal NPs composed by polyamidoamine (PAMAM) dendrimer were developed to escort cargo for cascade nuclear and bypass the intracellular drug resistance [98]. Part of primary amines in folic acid (FA) modified PAMAM polymers were amidized with an acid-labile β-carboxylic acid and also partly conjugated with (*S*)-(+)-camptothecin (CPT), to generate negatively charged FA-PAMAM/CPT conjugates. It could achieve multiple cascade intracellular delivery by following processes: (i) entering in cells via FA receptor-medicated endocytosis; (ii) rupturing the lysosome membrane and escaping into the cytoplasm by means of highly positive charge; and (iii) traversing to nucleus and releasing CPT quickly. On this occasion, this nuclear CPT could bypass the cell membrane-associated and cytosolic drug-resistance mechanisms, which can efficiently enhance its cytotoxicity (Figure 2).

### 4.2. Enhanced Drug Delivery to Cancer Stem Cell

The intratumoral heterogeneity of solid tumor, containing tumor cells, stroma, inflammatory infiltrates as well as complex vascular structures, presents a major barrier for effective drug delivery. Emerging evidence reveals that a small number of so-called cancer stem cells (CSCs), which could self-renew and give rise to non-tumorigenic daughter cells in tumor, could be the potential reason why conventional therapy cannot obtain satisfactory outcome, causing disease relapse and metastases [99,100]. Disappointingly, it is extremely difficult to kill and annihilate CSCs due to their high resistance to conventional chemotherapy and even radiotherapy [101]. Therefore, therapeutic approaches to disrupt the maintenance and survival of CSCs are currently imperative.

CSCs are commonly proposed to exist in a secondary niche within tumors that is more distant from the vasculature and more hypoxic. Recently, epithelial-like breast CSCs were found to be proliferative and located more centrally in the tumor [102]. Thus, free anticancer drugs cannot easily permeate into the tumor tissue and access into CSCs. Against to the conventional treatment strategies, NP-based drug delivery systems may possess significant advantages in the treatment of CSCs [103,104]. Nano-medicines may extravasate from tumor vessels and diffuse further and deeper into the tumor stroma, following to be internalized by CSCs. Recently, several application on nano-drugs for the selective treatment of CSCs have been explored, in the form of NPs enabled nucleic acid delivery vectors [105], and NPs-mediated hyperthermia [106,107]. However, there is not enough information currently available to make a conclusive statement regarding the therapeutic potential of these NPs, and the interaction between NPs and CSCs is not clear recently. Therein, the efficient internalization of drug-loaded NPs by CSCs was proposed to be a promising strategy for thorough CSCs elimination [108].

Accordingly, the enhanced effect of positive charge on the efficient cell-uptake of CSCs was evaluated [109]. Mesoporous silica nanoparticles (MSNs), modified positively charged with *N*-trimethoxysilylpropyl-*N,N,N*-trimethylammonium chloride (TMAC), were chosen to evaluate the uptake difference between positive- and negative-charge NPs in human mesenchymal stem cells (hMSCs). It appears that the cellular uptake of positively charged MSNs on hMSCs was highly efficient. Furthermore, the shift on surface charge of MSNs also altered its uptake mechanism. For unmodified MSNs, its uptake was involved in a clathrin- and an actin-dependent endocytosis behavior. However, the uptake in hMSCs of cationic MSNs was specific to be involved of positive surface charge, suggesting that positively-charged NPs possess higher internalization in CSCs.

**Figure 3:**
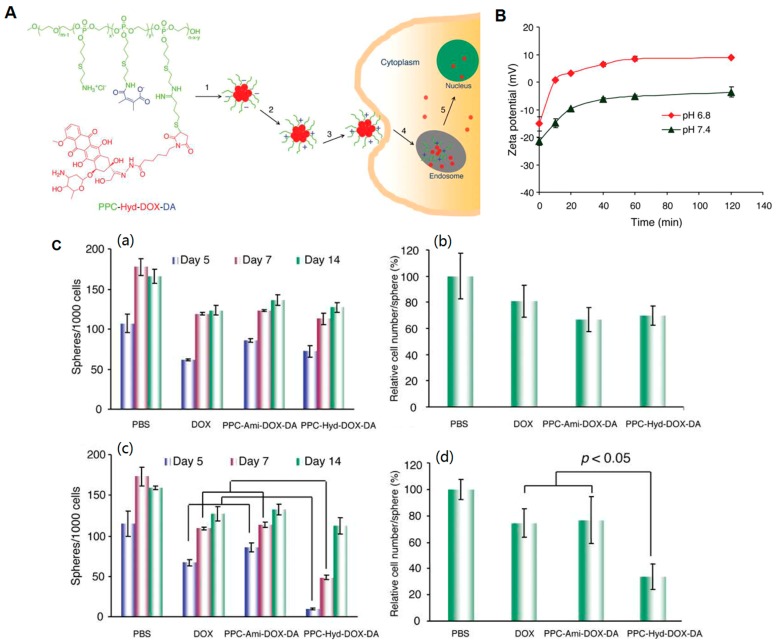
Tailor-made dual pH-sensitive charge-reversal polymer-doxorubicin NPs for cancer stem cell delivery: (**A**) schematic illustration of its pH-triggered cellular internalization; (**B**) zeta potential change of DOX conjugated on poly(ethylene glycol)-*b*-poly-(allyl ethylene phosphate), modified with cysteamine (PPC) and 2,3-dimethylmaleic anhydride (DMMA), by an acid-labile hydrazone bond (PPC-Hyd-DOX-DA) NPs at different pH value; and (**C**) time-dependent sphere formation of SK-3rd cells after incubation with various formulations (**a**,**c**) and the relative cell numbers/sphere measured at 14 days (**b**,**d**). Reproduced with permission from Du *et al.* (2011). Copyright 2011 ACS Publications.

Based on the faith that NPs with positive surface charge could benefit its transmission to CSCs, the activated charge-reversal approach would be certainly efficient to enhance the cellular uptake of CSCs and maintain sufficient intracellular drug concentration. A successful demonstration was provided by Du *et al.* [76], in which a pH-sensitive charge-reversal nano-medicine system was developed to inhibit drug resistance CSC (Figure 3). Briefly, a tailor-made dual pH-sensitive polymer-doxorubicin conjugate with PEG-polyphosphoester used as the polymer backbone, on which DOX was conjugated via a pH-sensitive hydrazone bond and DMMA was modified with the remaining amino groups, was synthesized (PPC-Hyd-DOX-DA). PPC-Hyd-DOX-DA NPs were negatively charged at pH 7.4 and shifted its zeta potential from negative to positive at pH 6.8 within 10 min. Given that the negatively-charged cell membranes, the cellular uptake of PPC-Hyd-DOX-DA NPs at different pH values in a drug-resistant cancer stem cell line SK-3rd should be variant. After 2 h incubation of SK-3rd cells with PPC-Hyd-DOX-DA NPs at pH 7.4, DOX cannot be delivered into SK-3rd cells efficiently due to its negatively charged feature. However, at pH 6.8, the number of spheres was remarkably reduced at days 5 and 7, indicating PPC-Hyd-DOX-DA NPs with acidic pHe stimulus could efficiently inhibit the self-renewal ability of CSCs *in vitro* and suppress its progression. This work demonstrated that the charge-reversal NPs could facilitate to inhibit the progression of CSCs.

### 4.3. Enhanced Multidrug Resistance Reversal

A major impediment to cancer treatment is multidrug resistance (MDR), including two elaborate categories: intrinsic and acquired [110,111]. The mechanism of MDR was involved in aberration in the genetic makeup of cancer cells by intrinsic or extrinsic factors like the long-term drug treatment. Specifically, MDR presents in the reduced uptake of hydrophilic drugs, the attenuation of cytotoxicity sensitivity of anticancer drugs, and the enhanced efflux of hydrophobic drugs by adenosine triphosphate (ATP)-binding cassette (ABC) transporters [112,113]. Recently, to reverse MDR by combining MDR inhibitors, for instance P-gp modulators, has been extensively employed, which was impossible to defeat all MDR mechanisms [114]. Meanwhile, the combination was hindered by low efficacy, high intrinsic toxicity, poor pharmacokinetics and biodistribution in tumor tissues [115]. An ancillary strategy using stimuli-responsive nanocarriers to append the already existing strategies is of great value to circumvent MDR in decades [116], even reaching into clinical trials [117]. However, these stimuli-responsive NPs still seem difficult to enter in tumor cells, showing a limited therapeutic response.

Actually, to deliver higher cytosolic concentration of anticancer drug would be one possible more general solution to overwhelm these common resistance mechanisms. Lee *et al.* [58] fabricated a charge-reversal micellar system (PHSM^pop-upTAT^) constitute of PLA_3k_-*b*-PEG_2k_-*b*-poly His_2k_-TAT and polyHis_5k_-*b*-PEG_3.4k_, mediated by pHe-trigged TAT shielding/deshilding to overcome MDR. This system exhibited the prolong circulation period and improved EPR effect due to a nearly neutral surface charge. When they reach into the tumor site, they can respond to pHe and expose TAT on surface of micelles by a pop-up behavior, turning into a positively charged form. TAT peptide could serve to quickly enter into mammalian cells, nonetheless whose confirmed internalization mechanism is the passive electrostatic interactions between polycationic TAT and anionic cells [118]. In this manner, the NP-cell membrane interaction and cellular uptake were significantly enhanced, following increasing the intracellular concentration of DOX loaded in micelles and improving its MDR reversal activity.

Four types of micelles including micelles with pH-sensitive TAT exposure (PHSM^pop-upTAT^), micelles with TAT exposure (PHSM^TAT^), micelles with pH-insensitive TAT exposure (PHIM^TAT^) and pH-insensitive micelles (PHIM) were prepared, respectively. For *in vitro* study, the improved cytotoxicity of PHSM^pop-upTAT^ by pH-triggered cationic TAT exposure was examined. Due to the over-expressed drug efflux pump of drug-resistant cells, free DOX exhibited lower cytotoxicity against NCI/ADR-RES cells than that against sensitive cells. Nevertheless, DOX/PHSM^pop-upTAT^ micelles at pH 7.0 and 6.8 showed 2.2- and eight-fold lower IC_50_ values than that at pH 7.4, respectively, because TAT peptide exposure-mediated endocytosis evades P-gp function. On the other hand, because of negative surface charge at all pH values, the PHIM^TAT^ and the PHIM shown poor cytotoxicity against drug-resistant cells. These results demonstrated that pHe-triggered TAT exposure resulting in positive surface charge of NPs facilitated the stronger interactions with tumor MDR cells and higher cytotoxicity against these cells.

As expected, this advantage was further confirmed in nude mice bearing human ovarian tumor drug-resistant A2780/AD cell. All DOX formulations including free DOX, DOX/PHSM^pop-upTAT^, DOX/PHSM, and DOX/PHSM^TAT^ were intravenously injected into tumor-bearing mice at a 3-day interval. After 3 weeks administration with 10 mg DOX/kg of body weight, the tumor growth regression activity of PHSM^pop-upTAT^ was greatly higher than other DOX formulations. Notably, although DOX/PHSM^TAT^ exhibited extremely high capacity to kill A2780/AD *in vitro* and *in vivo*, mice treated by DOX/PHSM^TAT^ showed about 10% loss, due to its non-specific uptake by normal cells. DOX/PHSM^pop-upTAT^ exhibited significant suppression on tumor growth in nude mice, in which the tumor size showed continuous reduction during the test period. This result indicated the significance of activated charge-reversal NPs against MDR, reducing systemic toxicity and specifically enhancing anticancer outcome.

## 5. Conclusions and Future Prospects

With the awareness of the correlation between intracellular drug concentration and anticancer efficiency, during the past decade increasing effects have been made in overcoming the internalization impediment with the aim of improving chemo-therapeutic outcomes. Recently, nanocarriers can overcome the shortcomings of *in vivo* delivery by prolonging retention time, enhancing tumor accumulation or achieving controlled drug release. However, the major problem hindering the application of nano-medicines is the insufficient cellular uptake by PEG. In this review, we provide an overview of the activated surface charge-reversal strategy in cancer treatment and highlight several representatives in enhancing cellular uptake, suppressing CSCs and overcoming MDR. Notably, because the precise entry pathways of CPPs are still controversial, the enhanced cellular uptake of CPPs-conjugated nanodrugs is mainly involved in the electrostatic interactions between its polycation and anionic cells. Thus, besides the conventional charge-reversal approaches of stimuli-responsive amides, the shielding/de-shielding transition of polycationic CPPs on surface of NPs was also categorized in this review. As a proof of concept, compared to their counterparts without charge-reversal activity, these activated charge-reversal NPs have shown significantly enhanced inhibition against cancer cells *in vitro* and *in vivo*. In general, this review clearly illustrates the potential and strength of activated charge-reversal nano-medicines for higher efficiency in overcoming cancer.

Nevertheless, it should be noted that, to develop charge-reversal NPs for cancer treatment, there is much room also for improvement. Firstly, either protonation of polymers or de-shielding of outer layer needs to be sensitive to unique physicochemical and biological features of tumor environment, resulting in reversing surface charge rapidly in tumor stroma or cytoplasm. This is a great challenge for polymer synthesis. Secondly, the effects of particle size on cellular uptake and biodistribution of NPs have been well elucidated. The stimuli-responsive de-shielding of outer shell would lead to remarkable size shrinkage. As a consequence, the influence of particle size change should be studied. Furthermore, more independent studies on the *in vivo* antitumor effect and pharmacokinetics of these activated charge-reversal NPs are needed. Stimuli-responsive charge-reversal nanocarriers provide new opportunities for more efficient cancer therapy.

## Figures and Tables

**Table 1:** Summary of stimuli-activated charge-reversal polymeric nanoparticles for cancer treatment.

Activation mechanism	Polymers	Reversal approach	Nano-carriers	Cargo	Test cells	References
pH-sensitive	polycaprolactone-*block*-PEI/amide-folic acid	20% of its primary and secondary amines in PEI converted into their amides	NPs	Doxorubicin	SKOV3	[48]
	d-α-tocopheryl polyethylene glycol 1000-poly-(β-amino ester) block copolymer containing disulfide linkages	Tertiary amine activated in acidic environment	NPs	Docetaxel	HepG2 and SMCC 7721	[52]
	poly(l-glutamic acid-*co*-l-lysine)	Manipulating the feed ratio of l-glutamic acid/l-lysine ratio	NPs	Cisplatin	HeLa	[54]
	poly(d,l-lactide)-*block*-poly(2-aminoethyl methacrylate)/DMMA	β-carboxylic amide produced by the reaction between amino on PAEMA and DMMA	Micelles	Doxorubicin	Hela	[72]
	poly(l-leucine)-block-Poly(l-lysine)/DMMA-TAT/succinyl chloride	β-carboxylic amide produced by the reaction between lysine amino and DMMA; TAT peptide masked by succinyl chloride	Micelles	Doxorubicin	Hela	[73]
	poly(2-aminoethyl methacrylate hydrochloride)-DMMA	Amides produced by the reaction between amino groups and DMMA	Nanogel	Doxorubicin	MDA-MB-435s	[49]
	biotin-poly(ethylene glycol) grafted poly(l-lysine)	Primary amine groups in the PLL backbone postmodified by citraconic anhydride	NPs	Quantum dot; Adenovirus	_	[74]
	poly(ε-caprolactone)-*block*-poly(allyl ethylene phosphate)-*graft*-2-(mercaptoethyl) trimethylammonium chloride/DMMA	pH-triggered amide bond	NPs	Doxorubicin	MDA-MB-231	[75]
	poly(ethylene glycol)-*b*-poly(allyl ethylene phosphate)/cysteamine-Hyd-DOX/DMMA	pH-triggered amide bond	Polymer-drug conjugate NPs	Doxorubicin	MDA-MB-231	[76]
	poly(l-aspartic acid)/DMMA	pH-triggered amide bond	Polypeptide NPs	Chlorin e6	B16F10, KB, MCF-7, A549, HeLa	[77]
	poly(l-lactic acid)-*b*-poly(ethylene glycol)-*b*-poly(l-lysine-Nε-(2,3-dimethyl maleic acid))	pH-triggered amide bond	Micelles	Doxorubicin	_	[78]
	1,5-dioctadecyl-l-glutamy-l,2-histidyl-hexahydrobenzoic acid	hexahydrobenzoic amide in HHG2C_18_	Liposomes	Temsirolimus	A498	[79]
	P(DMA-*co*-TPAMA)/PAH polyelectrolyte multilayers	The mixture of cationic and negatively charged polyelectrolyte	Polyelectrolyte -coated mesoporous silica NPs	Cisplatin; Rhodamine B	_	[80]
	c(RGDfK)-PEG-*b*-PLA/*N*-(3-aminopropyl)-imidazole -PEG-*b*-PLA	Imidazole (p*K*a = ~ 6.8) could achieve the transformation of protonation-deprotonation	Mixed micelles	Doxorubicin	EMT6	[81]
	PEG-*b*-C18	pH-dependent hydrolysis of benzoic-imine bond	Micelles	Doxorubicin	HepG2	[82]
	PEG-benzoic imine-poly(l-lysine)-cholic acid	pH-dependent hydrolysis of benzoic-imine bond	Micelles	_	Caco-2	[83]
	mPEG-ros-P(CL-*co*-DCL)	ROS sensitive thioether linkage; acid-labile β-carboxylic amides	NPs	Doxorubicin	HepG2 and L02 cell	[84]
	mPEG_2k_-*b*-polysulfadimethoxine_4k_/poly(l-histidine)_3.7k_-polyethyleneimine_1.8k_	charge shielding/deshielding	Mixed micelles	Paclitaxel	MCF-7;SKOV-3	[85]
	TAT-PEG-PLLA/poly(methacryloyl sulfadimethoxine) (PSD)-*b*-PEG	shielding/deshielding transition of TAT peptide	Mixed micelles	Doxorubicin	MCF-7	[57]
	poly(l-cystine bisamide-*g*-sulfadiazine)(PCBS)-*b*-PEG-TAT	shielding/deshielding transition of TAT peptide	Micelles	Doxorubicin	MCF-7	[86]
	mAb 2C5-PEG_3.4k_-PE/PEG_2k_-Hz-PE/TAT-PEG_1k_-PE	Deshielded TAT peptide by the clearance of pH-sensitive bond	Liposome	Doxorubicin	B16-F10, HeLa, MCF-7	[87]
	PLA_3k_-*b*-PEG_2k_-*b*-poly His_2k_-TAT/polyHis_5k_-*b*-PEG_3.4k_	pH-triggered “pop-up” of TAT	Micelles	Doxorubicin	A2780/Dox ^R^	[58]
	DMA-*N*-(2-hydroxypropyl) methacrylamide/*N*-(2-hydroxypropyl) methacrylamide-DoxR8 peptide	CPP R8 exposed by the clearance of DMA in acid environment	Polymer-drug conjugate NPs	Doxorubicin	Hela	[88]
Enzyme-sensitive	RGD-Hyaluronic acid coating polyethylenimine-poly(γ-benzyl l-glutamate)	Hyaluronic acid is used to shield the positive charges of PEI	Multiple layer NPs	DNA	Hela	[63]
	Polyethylenimine/Hyaluronic acid/Plasmid DNA	Hyaluronic acid is used to shield the positive charges of PEI	Ternary nano-complexes	Plasmid DNA	B16	[89]
	Hyaluronic acid coating R6H4 liposome	Hyaluronic acid is used to shield the positive charges of R6H4	Multiple layer liposome	Paclitaxel	HepG2	[90]
	Long-chain PEG-MMP2 substrate peptide-dextran-coated iron oxide	MMP2-responsive PEG	Multiple layer NPs	_	HT-1080	[91]
	PEG-MMP2 substrate peptide-QD	MMP2-responsive peptide linker	Multiple layer NPs	QDs	MDA-MB-435	[92]
	mAb 2C5-PEG_3.4k_-MMP2 peptide-DOPE/TAT-PEG_2k_-DSPE	MMP2-responsive PEG layer	Liposomes	_	4T1, H9C2	[67]
Redox-sensitive	DOPE-S-S-mPEG_5k_/DOPE-PEG_1.6k_-TAT	Shielded TAT could be exposed in higher GSH concentration	Liposomes	Calcein	HepG2	[68]
Thermo-sensitive	poly (*N*-iso-propylacrylamide) (PNIPAAm)	thermosensitive characteristics of PNIPAAm chain length to activate CPPs	Liposome	QDs	MDA-MB-435	[69]
	CPP-Dox/NGR-TSL	CPPs would be activated via heat stimulus	Liposome	Doxorubicin	HT-1080, MCF-7	[70]
UV-sensitive	1,2-distearoyl-sn-glycero-3-phosphoethanolamine-*N*-[maleimido(polyethylene glycol)-2000/1,2-distearoyl-SN-glycero-3-phospho-ethanolamine-*N*-[methoxy(polyethylene glycol)-2000	Reactivation of the peptide can be accomplished by releasing the constrain via UV-irradiation	Liposome	_	Hela	[71]
Thermo and pH dual-sensitive	poly(styrene-*co*-maleic anhydride)-*graft*-poly(2-(*N*,*N*-dimethylamino)ethyl methacrylate	(+) at 25 °C and pH 7.4; → (−) at 37 °C and pH 7.4; → (+) at 37 C and pH 6.8	Micelles	Doxorubicin	A2780/Dox ^R^	[93]

A2780/Dox ^R^ means DOX resistant ovarian A2780 cells.
